# Triptolide ameliorates colonic fibrosis in an experimental rat model

**DOI:** 10.3892/mmr.2015.3582

**Published:** 2015-04-01

**Authors:** QINGSONG TAO, BAOCHAI WANG, YU ZHENG, GUANWEI LI, JIANAN REN

**Affiliations:** 1 Department of Surgery, Affiliated Zhongda Hospital, Southeast University Medical School, Nanjing, Jiangsu 210089, P.R. China; 2 Department of Surgery, Research Institute of General Surgery, Jinling Hospital, Nanjing University Medical School, Nanjing, Jiangsu 210093, P.R. China

**Keywords:** triptolide, colonic fibrosis, inflammatory bowel disease

## Abstract

Triptolide is known to exert anti-inflammatory and immunomodulatory activities; however, its impact on intestinal fibrosis has not been previously examined. Based on our previous studies of the suppressive activity of triptolide on human colonic subepithelial myofibroblasts and the therapeutic efficacy of triptolide in Crohn’s disease, it was hypothesized that triptolide may have beneficial effects on intestinal fibrosis. In the present study, colonic fibrosis was induced in rats by 6 weekly repeated administration with a low-dose of 2,4,6-trinitrobenzene sulfonic acid (TNBS) and was then treated with triptolide or PBS daily (control) simultaneously. Extracellular matrix (ECM) deposition in the colon was examined with image analysis of Masson Trichrome staining. Total collagen levels in colonic homogenates were measured by a Sircol assay. Collagen Iα1 transcripts and collagen I protein were measured *ex vivo* in the isolated colonic subepithelial myofibroblasts by reverse transcription-quantitative polymerase chain reaction and immunoblot analysis, respectively. The results indicated that triptolide decreased ECM deposition and collagen production in the colon, and inhibited collagen Iα1 transcripts and collagen I protein expression in the isolated subepithelial myofibroblasts of the rats with colonic fibrosis. In conclusion, triptolide ameliorates colonic fibrosis in the experimental rat model, suggesting triptolide may be a promising compound for inflammatory bowel disease treatment.

## Introduction

Intestinal fibrosis is a common complication of inflammatory bowel disease (IBD) and occurs in ulcerative colitis (UC) and Crohn’s disease (CD) (1). Intestinal fibrosis is characterized by abnormal deposition of extracellular matrix (ECM) proteins produced by activated myofibroblasts, and is hypothesized to develop as a result of abnormal wound repair following local chronic inflammatory responses (2). Until now, few therapies have been shown to reliably treat intestinal fibrosis in IBD (3,4).

Subepithelial myofibroblast are important in these processes by regulating inflammatory responses and ECM metabolism (5,6). These generate a plausible link between mucosal inflammation and destruction of the subepithelial matrix. Inhibition of these processes represents a lucrative target for IBD anti-fibrosis therapies.

Triptolide, referred to as PG490, is the major active component of *Tripterygium wilfordii* Hook F (TWHF) extracts, has anti-inflammatory and immunomodulatory activities. Extracts of TWHF have been used in the treatment of glomerulonephritis and autoimmune diseases, such as rheumatoid arthritis and systemic lupus erythematosus (7,8). It has also been investigated as an immunosuppressant for kidney transplantation (9). A previous study showed that triptolide exerted antifibrotic effects in renal fibrosis (10), hepatic fibrosis (11) and lung fibrosis (12). However, the data of its effects on intestinal fibrosis caused by IBD remain to be elucidated. Our previous study showed that increased activation of chemokines interleukin-8 and monocyte chemoattractant protein-1 and matrix metalloproteinase (MMP)-3 expressed by human subepithelial myofibroblasts stimulated with pro-inflammatory cytokines could be inhibited by triptolide (13). In a cohort clinical trial, it was reported that triptolide could prevent the postoperative recurrence of CD (14). Recently, the therapeutic efficacy of triptolide in CD was also confirmed and it was shown that microscopic intestinal inflammation was attenuated with the modulation of *in situ* levels of inflammatory cytokines through the upregulation of Foxp3^+^ Tregs (regulatory T cells) (15). Based on these results, it was hypothesized that triptolide may have antifibrotic efficacy *in vivo* in chronic colitis with intestinal fibrosis through the therapeutic action against chronic inflammation. Therefore the present study aimed to evaluate the antifibrotic role of triptolide in rats with colonic fibrosis.

## Materials and methods

### Induction of colonic fibrosis

According to the described protocol (16), colonic fibrosis was established in male Sprague-Dawley rats weighing 1550200 g (Shanghai SLAC Laboratory Animal Co., Shanghai, China) by 6-weekly intrarectal instillation of increasing doses of TNBS (Sigma Chemical Co., St. Louis, MO, USA): 60, 60, 67.5, 67.5, 75, 75 mg/kg per week in 45% EtOH (Sigma). The rats were also administered 45 mg/kg per day of triptolide (PG490, molecular weight 360, purity 99%) intraperitoneally or phosphate buffered saline (PBS) starting with the initial TNBS treatment. Crystalline triptolide was obtained from the Institute of Dermatology, Chinese Academy of Medical Sciences (Nanjing, China).

At the time of tissue collection, the rats were sacrificed by carbon dioxide and the colons were removed intact from the anus to the ileocecal junction. Sections were taken from these regions for the following experiments: (i) Serial paraffin sections of the colon were stained with hematoxylin and eosin and Masson’s Trichrome to detect connective tissue. A pathologist examined each slide in a blinded manner. (ii) Isolation of subepithelial myofibroblasts. The present study was approved by the Institutional Animal Care and Use Committee for Southeast University Medical School and performed according to the institutional ethical guidelines stipulated by the Review Board for Southeast University Medical School.

### Image analysis of ECM content

The paraffin embedded blocks representing the similar positions of colon were sectioned and stained with Masson’s Trichrome. Quantitative digital morphometric analysis of ECM was performed according to a previously described method (17). In brief, 6–12 randomly selected fields for each section were photographed using a Spot digital camera (KY-F55MD; Olympus, Tokyo, Japan) and transformed into digital readings using Olympus Image Analysis software (Olympus Stream Ver.1.9.1; Olympus), which allowed for quantification of the various color wavelengths with pixels as the unit of measurement. The percentage of ECM was then calculated by dividing the pixel area of the ECM by the pixel area corresponding to the total tissue in the field of view.

### Sircol collagen assay

Total collagen content in the colon was detected with Sirius red collagen detection kit (Chondrex, Redmond, WA, USA). Colonic tissue was homogenized in T-PER buffer (Thermal Science, Amarillo, TX, USA) using a TissueLyser (Qiagen, Germantown, MD, USA), incubated on ice for 15 min, and centrifuged for 5 min at 10,600 × g at 4°C (Heraeus™ Primo™/Primo R centrifuge; Thermo Scientific, Waltham, MA, USA). Each protein sample was diluted in 0.5 M acetic acid to a final concentration of 100 *µ*g/ml. Optical density was read at 530 nm against the reagent blank using a DU-530 spectrophotometer (Beckman Coulter, Inc., Fullerton, CA, USA). Results were calculated based on collagen per 100 *µ*g/ml protein. Cultured SEMFs were characterized by immunohistochemistry. Mouse monoclonal antibodies against α-SMA, vimentin and desmin were used (Sigma). The cells were grown on glass coverslips and fixed using acetone, prior to immunoperoxidase staining with the Vectastain ABC peroxidase kit (Vecta Laboratories, Burlingame, CA, USA). Following incubation with the primary antibody, biotinylated goat anti-mouse immunoglobulin(Ig)-G (Sigma) was applied and subsequently avidin-biotinylated horseradish peroxidase complex. Peroxidase activity was developed with diaminobenzidine, followed by nuclear staining using hematoxylin (Sigma).

### Isolation and characterization of the colonic subepithelial myofibroblasts in the rats

We have previously established the isolation and primary culture of the colonic subepithelial myofibroblasts (13) according to the described protocol (18). In the present study, the method was slightly modified. Briefly, fresh mucosal samples were obtained from the colons at similar positions. After washing with calcium- and magnesium-free Hanks’ balanced salt solution (Gibco-BRL, Gaithersburg, MD, USA), the mucosa samples were completely denuded of epithelial cells by three 30-min incubations at 37°C in 1 mM EDTA (Sigma-Aldrich, St. Louis, MO, USA). The de-epithelialized mucosal samples were cultured in Dulbecco’s modified Eagle’s medium (Gibco BRL, Gaithersburg, MD, USA) containing 10% fetal bovine serum, 50 U/ml penicillin and 50 *µ*g/ml streptomycin (Gibco-BRL), and incubated at 37°C in a 5% CO_2_ atmosphere. The cells in suspension were removed after every 24- to 72-h culture period, and the denuded mucosal tissue was maintained in culture for up to 2 weeks. Established colonies of myofibroblasts possess the physiologic characteristics with immunostaining for α-smooth muscle actin (SMA) and vimentin. α-SMA is a contractile protein present in smooth muscle (19) and myofibroblasts (20). Vimentin is commonly used to stain myofibroblasts and fibroblasts. Desmin is an intermediate contractile filament that is a muscle specific protein (21). Intestinal myofibroblasts are immunoreactive for α-SMA as well as for vimentin, but completely negative for desmin. Myofibroblasts and fibroblasts or muscle cells also differ morphologically. Myofibroblasts tend to be spreading with numerous long processes that gave the cultures a complex overlapping appearance (Fig. 4). By contrast, muscle cells or fibroblasts appeared as elongated bipolar cells.

### Reverse transcription-quantitative polymerase chain reaction (RT-qPCR)

The mRNA expression of Collagen Iα1 (COL1A1) was determined by real-time polymerase chain reaction, as previously described (13). The cells were harvested with 0.25% trypsin (Sigma) and 0.02% EDTA, and total RNA was isolated using RNeasy reagents (Qiagen, Chatsworth, CA, USA), according to the manufacturer’s instructions. The mRNA concentration was quantitated by spectrophotometry (Beckman). For synthesization of cDNA, 1 *µ*g total RNA was treated with reverse transcriptase (Promega, Madison, WI, US) and oligo (dT) were used for reverse transcription. The reactions were performed using the Reverse Transcription system (Promega) under the following conditions: 42°C for 15 min, 95°C for 5 min and 4°C for 5 min. Samples were stored at −20°C until use.

qPCR analysis was performed using an ABI PRISM 7700 (Perkin-Elmer, Applied Biosystems, Foster City, CA, USA). Specific primers and dual-labelled fluorescent probes were designed using the Primer Express primer design program v1.01 (Perkin-Elmer). The constitutively expressed GAPDH was used as an internal control. Probes were labeled with the fluorescent reporter dye 5-carboxyfluorescein (FAM) at the 5′ end and the quencher N,N,N,N′-tetramethyl-6-carboxyrhodamine (TAMRA) or Minor Groove Binder (MGB) at the 3′ end. The primer and probe sequences were as follows: Collagen Iα1, forward 5′-AATCAGCCGCTCCCATTCTCCTA-3′, reverse 5′-GGAGGGCGAGGGAGGAGAGAA-3′ and probe 5′-(FAM)-TCATCCCGCCCCCATTCCCTG-(MGB)-3 and GAPDH, forward 5′-GGCAAATTCAACGGCACAGT-3′, reverse 5′-AGATGGTGATGGGCTTCCC-3′ and probe 5′-(FAM)-AAGGCCGAGAATGGGAAGCTTGTCATC -(MGB)-3′. The samples were amplified in a final volume of 25 *µ*l. The primers were used at a concentration of 900 nM, and probes at 250 nM. GAPDH was amplified in separate reactions. The cycling conditions were as follows: 50°C for 2 min, 95°C for 10 min, 45 cycles of 95°C for 30 sec and 60°C for 30 sec. The data were normalized to GAPDH gene expression and are expressed as fold increase in expression

### Western blot analysis

The activity of Collagen I was determined by western blotting. The cells were harvested and sonicated in solubilization buffer, containing 20 mM Tris-HCl, (pH 8.0), 150 mM NaCl, 1 mM EDTA, 1 mM EGTA, 1% Triton X-100, 2.5 mM sodium pyrophosphate, 1 mM sodium vanadate, 10 *µ*g/ml aprotinin, 10 *µ*g/ml leupeptin, 1 mM phenylmethylsulfonyl fluoride. The cell debris was removed by centrifugation at 10,000 × g for 15 min and the supernatants were boiled in Laemmli sample buffer (Bio-Rad) for 5 min. An equal quantity of protein was subjected to sodium dodecyl sulfate-10% polyacrylamide gel electrophoresis, and the proteins were blotted onto a PVDF membrane (Amersham Pharmacia Biotech, Piscataway, NJ, USA). The membranes were blocked with 5% skimmed milk in Tris-buffered saline, containing 0.1% Tween 20 (pH 7.6), overnight at 4°C and were probed with primary rabbit antibodies (Abcam, Cambridge, MA, US) for 1 h at room temperature. Following washing, the membranes were incubated with secondary goat anti-rabbit antibody (Abcam) coupled to horseradish peroxidase for 1 h at room temperature. Antibody-antigen complexes were then detected using an ECL chemiluminescent detection system (Amersham Pharmacia Biotech). Quantification was performed by densitometry.

### Statistical analysis

Statistical significance was determined by a t-test or analysis of variance followed by Fisher’s least significant difference post hoc test, as appropriate, using SPSS V20 (IBM, Armonk, NY, USA). Data are expressed as the mean ± standard error. P<0.05 was considered to indicate a statistically significant difference.

## Results

### Triptolide decreases ECM deposition in vivo in the colon

**Figure 1:**
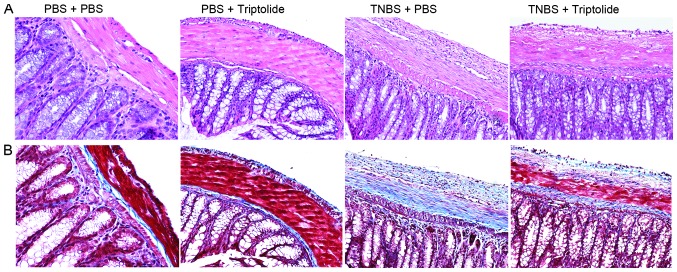
Image analysis of the colonic tissue. (A) Hematoxylin and eosin staining (magnification, x20). Hyperemia, colonic crypt hyperplasia, and inflammatory cells infiltration were reduced with triptolide treatment. (B) Masson Trichrome staining (magnification, x20). Extracellular matrix deposition (blue area) was decreased with triptolide treatment. PBS, phosphate-buffered saline; TNBS, 2,4,6-trinitrobenzene sulfonic acid.

**Figure 2:**
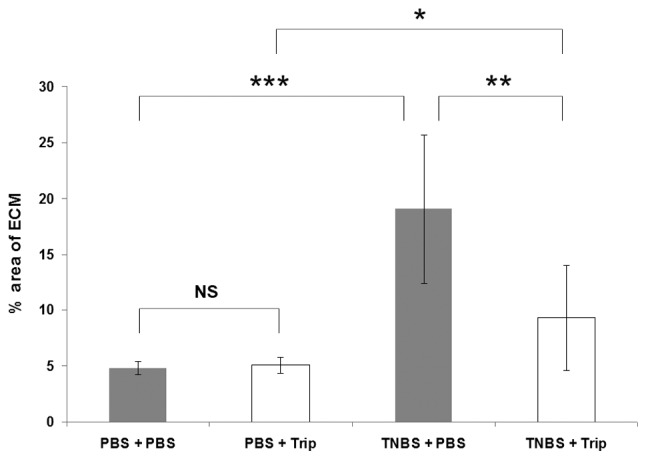
Image analysis of quantified ECM deposition in the colon. Triptolide treatment reduced ECM deposition. Results are expressed as the mean ± standard error for 10 rats/group. ^*^P<0.05; ^**^P<0.01 and ^***^P<0.001; NS, no significance; Trip, triptolide; ECM, extracellular cellular matrix; TNBS, 2,4,6-trinitrobenzene sulfonic acid.

At the endpoint of the study (42 days post induction), in the TNBS-treated rats without triptolide treatment, severe intestinal fibrosis and stricture were found particularly in the distal 5 cm of the colon. The lamina propria showed pockets of inflammation alternating with areas of minimal or moderate inflammation as well as persistent edematous swelling of the colonic wall (Fig. 1A). Furthermore, the colon exhibited marked collagen deposition in the subepithelial and serosal areas determined with Masson Trichrome staining (Fig. 1B). By contrast, the rats in the TNBS/triptolide-treated group exhibited less severe inflammatory changes and only developed slight colonic fibrosis. As shown in Fig. 2, the area of ECM deposition quantified from the ‘blue area’ stained with Masson Trichrome was similar: 4.8±0.6 and 5.1±0.7% in the PBS/PBS and PBS/triptolide-treated rats, respectively. The area of ECM deposition was 19±6.6% in the TNBS/PBS-treated rats (P<0.001, vs. the PBS/PBS-treated rats). It decreased to 9.3±4.7% in the TNBS/triptolide-treated group, and was significantly lower than the TNBS/PBS-treated group (P<0.01). No intestinal inflammation or fibrosis was found in the PBS-treated mice with or without triptolide treatment as expected.

### Triptolide decreases total collagen production in vivo in the colon

**Figure 3:**
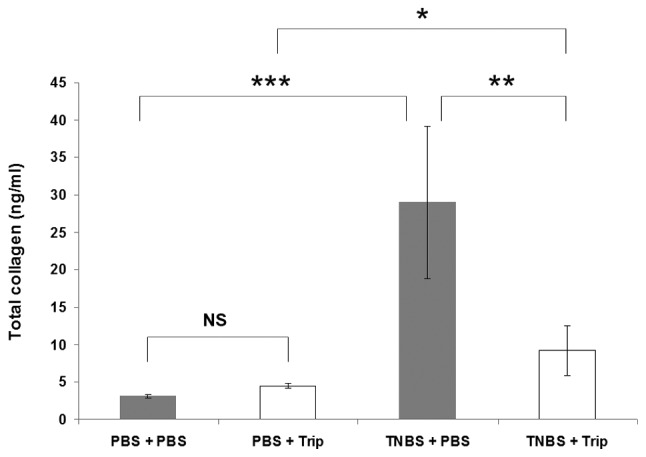
Total collagen levels in colonic homogenate were measured by a Sircol assay. Triptolide treatment decreased total collagen levels. Results are expressed as the mean ± standard error for 10 rats/group. ^*^P<0.05; ^**^P<0.01 and ^***^P<0.001. NS, no significance; Trip; triptolide; TNBS, 2,4,6-trinitro-benzene sulfonic acid.

As shown in Fig. 3, total collagen production in the colon of the PBS/PBS-treated rats measured with the Sircol assay was 3.1±0.23 ng/ml, and it increased to 29±10.1 ng/ml in the TNBS/PBS-treated rats (P<0.001). In the PBS/triptolide-treated rats, total collagen expression was 4.5±0.34 ng/ml, and it increased to only 9.2±3.3 ng/ml in the TNBS/triptolide-treated rats; however, there was a significant difference between the two groups (P<0.05). Compared with the TNBS/PBS-treated rats, total collagen expression in the colon of the TNBS/triptolide-treated rats significantly decreased (P<0.01).

### Characterization of the colonic subepithelial myofibroblasts

**Figure 4:**
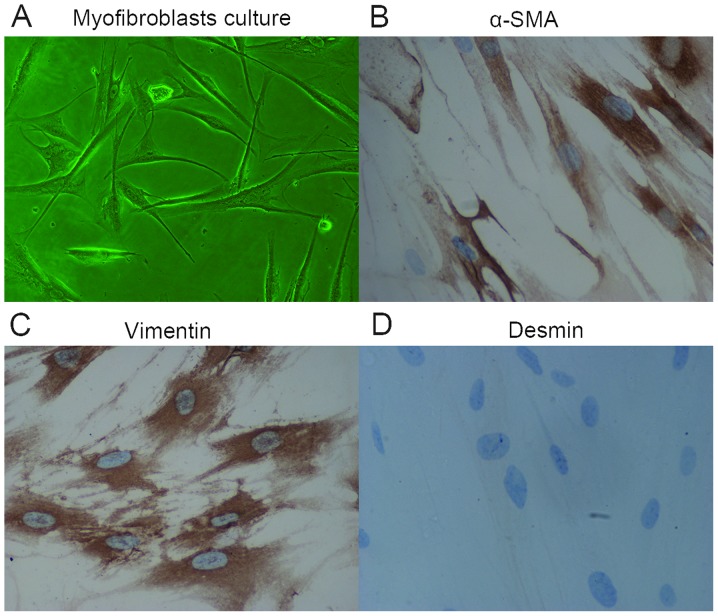
Characterization of isolated subepithelial myofibroblasts. (A) Images of isolated myofibroblasts (magnification, x100). Immunostaining with monoclonal antibodies. (B) Positive staining for α-SMA; (C) positive staining for vimentin; and (D) negative staining for desmin. Magnification, x400. α-SMA, α-smooth muscle actin.

Intestinal myofibroblasts are immunoreactive for α-SMA and vimentin, however, completely negative for desmin. Myofibroblasts and fibroblasts or muscle cells also differ morphologically. Myofibroblasts tended to be spreading with numerous long processes, which gave the cultures a complex overlapping appearance (Fig. 4).

### Triptolide suppresses collagen Iα1 mRNA and collagen I protein expression in the colonic subepithelial myofibroblasts isolated from the rats with chronic colitis and colonic fibrosis

**Figure 5:**
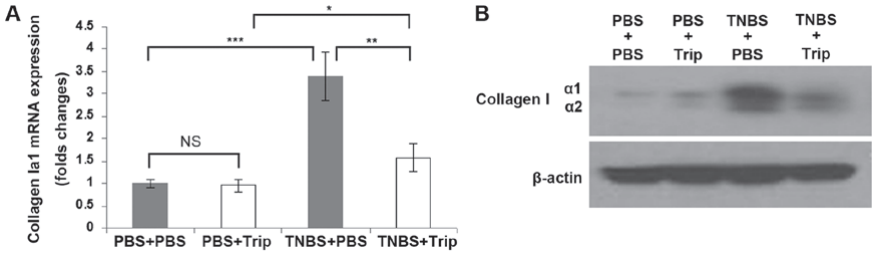
Effect of triptolide *ex vivo* in the isolated subepithelial myofibroblasts from the colon of rats with chronic colitis and colonic fibrosis. (A) Collagen Iα1 transcripts in isolated colonic subepithelial myofibroblasts were measured by reverse transcription-quantitative polymerase chain reaction. Results are expressed as the mean ± standard error for the samples from 10 rats/group. ^*^P<0.05; ^**^P<0.01 and ^***^P<0.001. (B) Collagen I protein in isolated colonic subepithelial myofibroblasts was measured by immunoblot analysis. Results are from the representative experiment. NS, no significance; PBS, phosphate-buffered saline; Trip, triptolide TNBS, 2,4,6-trinitrobenzene sulfonic acid.

Collagen Iα1 mRNA expression was measured *ex vivo* in the subepithelial myofibroblasts (Fig. 4) isolated from the rat colon. Collagen Iα1 mRNA expression increased to 3.4±0.54 fold in the TNBS/PBS-treated rats compared with the PBS/PBS-treated rats (P<0.001, Fig. 5A). By contrast, collagen Iα1 mRNA expression in the TNBS/triptolide-treated rats decreased to only 1.57±0.30 fold that of the PBS/PBS-treated rats (P<0.05), and only 1.63±0.45 fold that of the PBS/triptolide-treated rats (P<0.05). Compared with the TNBS/PBS-treated rats (3.4±0.54), the expression of collagen Iα1 in the TNBS/triptolide-treated rats (1.57±0.30) was significantly lower (P<0.01). As shown in Fig. 5B, similar results were obtained, when collagen I protein was measured by immunoblot analysis.

## Discussion

In the present study, the antifibrotic effect of triptolide in rats with colonic fibrosis was examined. It was found that repeated treatment with low-doses of TNBS induced colonic fibrosis. Triptolide reduced ECM deposition and collagen production *in vivo* in the colon, and decreased *ex vivo* collagen Iα1 mRNA and collagen I protein production in the colonic subepithelial myofibroblasts.

After a 6-week induction of low-dose TNBS, there were no obvious acute colitis signs, such as ruffled coats, hunched posture or restricted movement as the model used in these studies is not a model of acute inflammation, but chronic inflammation and colonic fibrosis. This experimental model of colonic fibrosis mimics the process of chronic stricture formation and progression which occurs in IBD. Once this process of fibrosis is initiated in the susceptible patient, it is self-perpetuating and chronic inflammation, instead of acute inflammation was induced in this experimental model of colonic fibrosis (22,23).

Excess extracellular matrix production is central to the aberrant wound healing process leading to fibrosis in a number of organs, including the intestine (24). IBD is a chronic, progressive disease of the gastrointestinal tract with an unknown etiology and CD is characterized by transmural inflammation of all layers of the bowel wall (25). The inflammatory changes in intestinal physiology result in the majority of the symptomatology associated with CD (26) and significant morbidity results from the irreversible tissue injury and fibrosis that frequently occur in chronically inflamed bowel segments (25,27). For reasons unknown, the reparative process associated with CD can progress uncontrollably, leading to enhanced proliferation along with defective programmed cell death of mesenchymal cells, and the unrestrained deposition of ECM (28,29). The recurrence of fibrosis is the predominant reason for obstruction, however, few therapies have reliable effect on the inhibition of fibrosis (30–33). To the best of our knowledge the present study is the first to report that triptolide diminishes ECM deposition in rats with colonic fibrosis. This may be a potential mechanism by which triptolide protects the intestine under inflammatory conditions and act as a therapeutic agent for treatment of IBD (14).

Triptolide has been demonstrated to possess anti-inflammatory and immunosuppressive effects (34,35). Triptolide inhibits lymphocyte proliferation, synthesis and secretion of proinflammatory cytokines (36,37), and also induces apoptosis in T cells and lymphoma cell lines (38,39). In addition, triptolide inhibits dendritic cell-mediated chemoattraction of neutrophils and T cells by inhibiting Stat3 phosphorylation and nuclear factor-κB activation (39). We previously reported that mRNA and protein expression of chemokines, including interleukin (IL)-8 and monocyte chemotactic protien (MCP)-1, and stromelysin-1 in colonic subepithelial myofibroblasts were inhibited *in vitro* by triptolide in a similar manner (13). Healthy intestinal mesenchymal cells produce limited amounts of collagen and fibronectin, which serve to maintain tissue integrity and facilitate healing. On exposure to chronic inflammation, mesenchymal cells transform into activated myofibroblasts, a pro-repair and pro-fibrogenic cell phenotype, and markedly increase the production of ECM (40,41). In addition, the finely tuned balance between tissue matrix metalloproteinases, which degrade ECM, and tissue inhibitors of metalloproteinases, which inhibit ECM degradation, is lost, promoting further structural changes of the colonic bowel wall (42,43). In the present study, decrease in the extent of intestinal fibrosis following treatment with triptolide may be due to downregulation of collagen expression in the colonic subepithelial myofibroblasts.

Triptolide has several characteristics of particular interest in relation to IBD (44). Our previous study demonstrated that increased activation of chemokines, including IL-8 and MCP-1, and matrix metallo proteinases-3 expressed by human subepithelial myofibroblasts stimulated with pro-inflammatory cytokines could be inhibited by triptolide (45). In a cohort clinical trial, we reported that triptolide prevented the postoperative recurrence of CD (46). Recently, we also confirmed the therapeutic efficacy of triptolide in CD and demonstrated that microscopic intestinal inflammation was attenuated with the modulation of *in situ* levels of inflammatory cytokines through the upregulation of regulatory T cells (47). It inhibits several proinflammatory cytokines and adhesion molecules, which are all important mediators of IBD (48,49). Triptolide has been shown to be safe and clinically beneficial in rheumatoid arthritis (49). It has also been shown to be effective in the treatment of several autoimmune diseases, such as lung fibrosis (12) and uveoretinitis (50) in animal models. Yan *et al* (51) showed triptolide could inhibit IFN-γ-induced activation of fibroblasts derived from patients with Graves’ ophthalmopathy. We previously reported that triptolide could prevent the postoperative recurrence of CD in a clinical trial (14). Steroids have been administered widely for their anti-inflammatory activity in IBD, but they are not free of adverse effects. Such adverse reactions may be avoided if triptolide proves effective for the treatment of IBD, particularly for CD. The present study indicated that triptolide may be a potential therapeutic agent for IBD due to its extracellular matrix protective and anti-inflammatory properties. Whether triptolide is a viable adjunctive for treatment of IBD and is devoid of adverse effects remain to be clarified. Further studies are required to understand the underlying mechanisms and potential limitations of treatment.

In conclusion, inhibition of colonic fibrosis by treatment with triptolide in the experimental rat model may suggest triptolide as a promising compound for IBD treatment.
